# ETE: a python Environment for Tree Exploration

**DOI:** 10.1186/1471-2105-11-24

**Published:** 2010-01-13

**Authors:** Jaime Huerta-Cepas, Joaquín Dopazo, Toni Gabaldón

**Affiliations:** 1 Comparative Genomics group, Bioinformatics and Genomics Programme, Centre for Genomic Regulation (CRG), Dr. Aiguader, 88 08003 Barcelona, Spain; 2 Bioinformatics Department, Centro de Investigación Príncipe Felipe, València, Spain

## Abstract

**Background:**

Many bioinformatics analyses, ranging from gene clustering to phylogenetics, produce hierarchical trees as their main result. These are used to represent the relationships among different biological entities, thus facilitating their analysis and interpretation. A number of standalone programs are available that focus on tree visualization or that perform specific analyses on them. However, such applications are rarely suitable for large-scale surveys, in which a higher level of automation is required. Currently, many genome-wide analyses rely on tree-like data representation and hence there is a growing need for scalable tools to handle tree structures at large scale.

**Results:**

Here we present the Environment for Tree Exploration (ETE), a python programming toolkit that assists in the automated manipulation, analysis and visualization of hierarchical trees. ETE libraries provide a broad set of tree handling options as well as specific methods to analyze phylogenetic and clustering trees. Among other features, ETE allows for the independent analysis of tree partitions, has support for the extended newick format, provides an integrated node annotation system and permits to link trees to external data such as multiple sequence alignments or numerical arrays. In addition, ETE implements a number of built-in analytical tools, including phylogeny-based orthology prediction and cluster validation techniques. Finally, ETE's programmable tree drawing engine can be used to automate the graphical rendering of trees with customized node-specific visualizations.

**Conclusions:**

ETE provides a complete set of methods to manipulate tree data structures that extends current functionality in other bioinformatic toolkits of a more general purpose. ETE is free software and can be downloaded from http://ete.cgenomics.org.

## Background

Trees are commonly used to represent the results of many bioinformatics analyses. In particular, such type of binary graphs are ideal to describe the hierarchical relationships among a variety of biological entities. Some common examples are the evolutionary analysis of molecular sequences or the clusterization of genes and proteins according to their properties. Besides the information encoded in the topology of trees, branch lengths can also be scaled to provide information on the distances between the different partitions. In phylogenetics, for instance, trees are used to illustrate the evolutionary relationships among species or molecular sequences, considering terminal nodes as extant Operational Taxonomic Units (OTU) and internal nodes as their corresponding ancestors. In such phylogenetic trees, branch lengths are usually proportional to the evolutionary distance among sequences. Other applications, such as the analysis of gene expression, use hierarchical clustering analysis to group genes or experimental conditions according to the similarity of their expression patterns. Likewise, trees are used by many protein classification methods and for the analysis of phylogenetic profiles. Thus, the analysis of tree data structures is a common task in many areas of bioinformatics and there is a need for analytical and visualization tools. In this respect, a number of bioinformatic programs do exist that assist in the exploration of hierarchical trees. Most of them, however, consist of standalone applications that are focused on visualization and, occasionally, on performing specific tests. Some well known examples are TreeView [1], a widely used program for inspecting phylogenetic trees; Cluster Treeview [2], an application for visualizing microarray clustering results; ATV [3], a java program used to explore phylogenies which provides also some editing options; MEGA [4], an evolutionary genetics analysis suite that includes a built-in tree viewer; and many other recent applications [5-8]. While all these programs are very useful to manage single trees, they can hardly be automatized or adapted to specific needs. Thus, when the analysis of hundreds or thousands of trees is required, the use of standalone programs becomes restrictive, because a much higher level of automation is required. In such cases, programming toolkits represent a more adequate framework, since they provide tools and methods to handle data at a lower level. Using toolkits, bioinformaticians can easily create their own analysis pipelines and program custom tasks over large collections of data [9]. Several generic bioinformatic toolkits do exist that cover a wide range of programming languages and scopes, with BioPerl [10] and BioPython [11] being the most extensively developed. Together with a broad range of other features, these toolkits allow certain level of interaction with tree data structures. However, only basic actions are currently supported. Alternatively, the PyCogent [12] and P4 http://bmnh.org/~pf/p4.html python toolkits can be used to extend this functionality, although they are mostly focused on phylogenetic reconstruction. R [13], a general purpose statistical framework, does include several packages to perform statistical tests on clustering and phylogenetic trees. Nevertheless, these packages are focused on performing specific analyses rather than in providing tree handling and manipulation features. Finally, in contrast to the great number of standalone tree viewers, programming toolkits offer few, if any, graphical rendering possibilities. An intermediate alternative between standalone viewers and programmatic tree rendering is that of the TreeDyn program [14], which has support for some scripting options and can be used to create fully annotated tree images.

In response to these limitations, we present here the Environment for Tree Exploration (ETE), a python programming toolkit to analyze, manipulate or visualize any kind of hierarchical tree. It extends the functionality in other toolkits and allows a high level of customization. ETE's drawing features, although less exhaustive than in standalone editors, rely on the Python scripting language, which makes possible to combine advanced tree analyses and tree visualization into a single program. The toolkit includes methods to browse and manipulate tree topologies, provides support for the New Hampshire eXtended (NHX) format and allows advanced actions such as node annotation, automatic rooting, cut & paste partitions, tree concatenation, node search, and branch distance related operations. In addition, ETE implements two specific modules to work with phylogenetic and clustering trees. The phylogenetic extension allows trees to be linked to their corresponding multiple sequence alignments, includes two orthology and paralogy prediction algorithms, implements the duplication dating method described in [15] and provides access to the PhylomeDB database [16]. Similarly, clustering trees can be linked to their source data, which allows tree partitions to be analyzed through several validation techniques. Additionally, ETE implements a fully programmable drawing engine that can be used to generate, dynamically, custom tree representations in PDF or PNG formats. This drawing engine is fully integrated with the built-in extensions, thus providing pre-defined visualization layouts for clustering trees and phylogenies. A Graphical User Interface is also included which allows on the fly interaction with trees.

Currently, the ETE toolkit is used in diverse projects, including GEPAS [17], Phylemon [18] and PhylomeDB [15]. ETE package and documentation can be accessed at http://ete.cgenomics.org

## Implementation

ETE is entirely written in Python [19], a programming language that offers a strong support for integration with other languages and tools, and whose popularity is raising among the bioinformatics community [20]. ETE's philosophy is to facilitate the integration with other toolkits as well as to provide a scalable program architecture. Thus, ETE tree objects can be easily imported and expanded by incorporating custom methods and properties. The functionality of the ETE toolkit is divided into several python modules, which can be imported at convenience. A summary of features of the different modules is shown in Table 1.

**Table 1:** Summary of ETE's features

Module	Description
General trees	Advanced node annotation, tree topology manipulation, automatic tree pruning, cut & paste partitions, trees concatenation, random trees generation, iterate over leaves and descendants, pre and pos-order tree traversal, rooting options, advanced nodes search, get distances among nodes, detect midpoint outgroup, find farthest descendant node, find farthest node in the whole tree, detect first common ancestor among nodes, text mode visualization, newick rendering (several formats), extended newick format integration, built-in python operations: print, len, iter, in.

Phylogenetic trees	Link to multiple sequence alignments, automatic species name detection, check node monophyly, evolutionary events dating, detect orthology and paralogy relationships: species overlap and tree reconciliation methods, complete access API to the phylomeDB database, integrated visualization (show molecular sequences and evolutionary events).

Clustering trees	link to numerical matrices, calculate inter and intra-cluster distances among clusters, calculate Silhouette and Dunn Indexes, integrated visualization (display numerical profiles in several formats).

Tree Visualization	Interactive Graphical User Interface, programmable drawing engine, independent node aspect editing, support drawing node extra features (text or external images), vector graphics rendering using PDF format.

## Results

### Tree handling module

ETE's main module allows to read and render trees using the two most common formats: New Hampshire (NH) and New Hampshire eXtended (NHX). Moreover, it allows to generate random trees or create custom tree structures from scratch. In order to increase compatibility with other tools, several newick format standards are currently supported by ETE, both for reading and writing trees (see ETE's extended documentation). ETE's trees are internally encoded as a series of tree node instances connected following a parent-child relationship. Each node is encoded as an independent Python object, which provides many methods to manipulate its connections (i.e. add, remove, delete or detach nodes) and to easily browse its topology (i.e. tree traversal and get terminal, children, sister or descendant nodes). As a consequence, each internal node is treated as a fully featured subtree, thus allowing to analyze different parts of trees separately. ETE's tree object implementation supports multifurcations and can be used to deal with very large structures. As a reference, the NCBI taxonomy newick tree file, with more than 450.000 nodes, could be loaded as an ETE tree object in 40 seconds (Intel Xeon CPU 2.33 GHz).

One of the main advantages of ETE as compared to other toolkits is that nodes can contain additional information other than topology and branch distances. Users are free to associate any external data to the different tree nodes and then use such data in the subsequent analyses, integrate them as part of their tree images or save them using the NHX format. This functionality provides the possibility of creating fully annotated trees.

Finally, a complete set of operations are available to browse, analyze or modify trees: i.e.) pre- and post-order traversing strategies, search nodes or partitions matching specific criteria, midpoint and species-guided automatic tree rooting, calculate distances between branches, detection of common ancestors, random tree building, topology manipulation, tree pruning, tree concatenation, cut and paste partitions, etc.

### Tree rendering and visualization

The visualization module provides a programmable drawing system to render hierarchical tree structures as dendrograms. The core of this extension consists of an image drawing algorithm that can be controlled by a set of user-defined rules. Such rules can be written as small python functions and are used to determine, dynamically, the aspect of each tree node. This allows, for instance, to vary the aspect of each node according to its internal properties. Moreover, the rendering engine allows not only to draw tree topologies but also to incorporate external graphical information to each node. Thus, external images, graphs or custom text labels can be added to nodes as part of the general tree image. The programatic use of the tree drawing module allows users to control how trees are rendered and what information is shown for the different nodes. Images can be rendered as PNG or PDF files.

In addition to the drawing engine, trees can be interactively visualized using a built-in Graphical User Interface (GUI), which allows on the fly manipulation of trees. Thus, each tree node has its own displaying method that can be used to start the visualization of its specific topology. The GUI is fully integrated with other ETE's features and allows to interact with tree nodes and their properties in a graphical way. Both the GUI and the rendering engine use the last Qt4 programming libraries to increase the performance of large images visualization. Qt4 is available for GNU/Linux, MacOS and Windows, and it is distributed as free software. A complete description of the usage of this module, as well as examples, can be found within the ETE tutorial at http://ete.cgenomics.org.

### Phylogenetic extension

**Figure 1:**
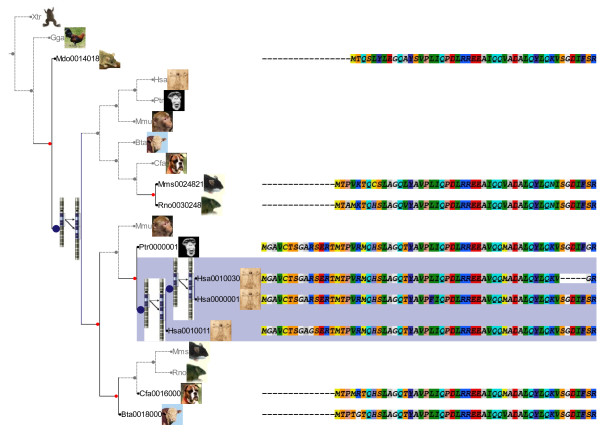
**Screenshot of a reconciled phylogenetic tree displayed with ETE**. The image shows a portion of a reconciled tree generated using the ETE's strict reconciliation algorithm over an example gene phylogeny. The most accepted tree of life for the species considered was used for reconciliation. A custom ETE layout function has been used to highlight different aspects of the tree: Grey dashed lines represent the inferred gene losses. Blue nodes indicate duplication events. Red nodes represent speciation events. Each OTU is displayed with their corresponding name and species image. Part of the multiple sequence alignment is displayed with each sequence associated to its corresponding OTU.

Phylogenetic trees are the result of most evolutionary analyses. ETE's phylogenetic extension implements a special type of tree instances which are focused on the analysis of evolutionary trees. Thus, tree leafs are considered OTUs, while internal nodes are considered their ancestors. By applying different strategies, ETE can automatically assign internal nodes to speciation and duplication events, thereby establishing orthology and paralogy relationships between the OTUs [21]. Two built-in methods are available to predict speciation and duplication events: a species-overlap algorithm that is independent of the species tree [15] and the classical gene-tree/species-tree reconciliation algorithm [22]. Both methods return a list of orthology and paralogy predictions and annotate the original tree nodes according to the detected evolutionary events. Additionally, nodes predicted as gene duplication events can be dated using the topology scanning method described in [15]. Furthermore, molecular phylogenies can be associated with their corresponding multiple sequence alignments, thus establishing a link between each tree leaf and its sequence. This allows the visualization of aligned sequences together with the tree topology, which helps to detect, for instance, linage-specific changes, specific domain composition or conserved regions. As sequences are considered an additional property of tree leafs, they can be combined with the tree browsing capabilities to program analyses such as detecting clades with certain level of sequence conservation or composition. This extension is fully integrated with the drawing module, and includes a pre-defined visualization layout to explore trees together with its predicted evolutionary events and the sequences associated to nodes (Figure 1). Other features such as checking the monophyly of nodes, the automatic detection of species names, or the guided selection of outgroups are also available.

### PhylomeDB API

PhylomeDB [16]http://phylomedb.org is a database for complete collections of gene phylogenies (phylomes). It currently stores more than 420,000 trees and 120,000 multiple sequence alignments reconstructed using a high-quality phylogenetic pipeline, which includes Maximum Likelihood or Bayesian tree inference, alignment trimming and evolutionary model testing.

ETE provides a complete Application User Interface (API) for accessing phylomeDB. Throughout this API, users can connect to the phylomeDB database and search for pre-computed gene phylogenies, download complete phylomes or obtain the orthology and paralogy predictions provided by the database. The phylomeDB API is fully integrated with the ETE software and trees can be automatically downloaded as phylogenetic tree instances.

### Microarray clustering trees

Microarray expression data are usually encoded as matrices in which rows represent the expression profile of genes across different conditions (columns). A variety of clustering analyses are used to group genes that respond coordinately to a given set of conditions or, conversely, to group conditions according to their gene expression profiles. In such cases, genes are generally represented by the terminal tree nodes whereas internal nodes represent different levels of similarity among the expression profiles of grouped genes.

**Figure 2:**
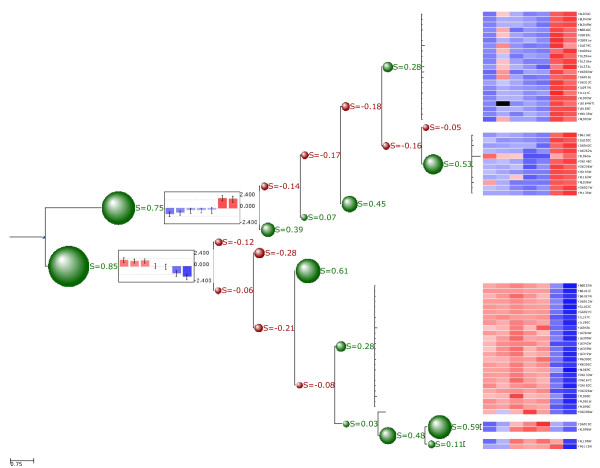
**Screenshot of a microarray clustering tree validated and displayed with ETE**. Expression profiles of terminal nodes (genes) are shown as a heatmap at the right side of the image. Silhouette indexes were calculated for each internal partition and are displayed in the tree. Size of green and red bubbles is proportional to the silhouette value. Red bubbles stand for silhouette values lower than 0 and green bubbles for silhouettes higher than 0. Two graphs representing the mean expression profile of the two most basal tree branches are also included in the image.

ETE's clustering extension can be used to import microarray clustering results and link them to their corresponding gene expression patterns. Thus, the expression profile of any internal tree node (cluster) can be calculated as the mean expression pattern of the grouped leaves (usually representing genes). This allows, for instance, to automate the detection of co-expressed genes in large datasets or to find nodes matching certain expression patterns. Moreover, several clustering validation techniques are implemented that allow to evaluate the goodness of fit of the different tree partitions. Currently, inter- and intra-cluster distances, cluster standard deviation, the Dunn index [23] and the cluster Silhouette index [24] can be calculated. Similarly to the phylogenetic extension, several visualization layouts are also provided for displaying clusters together with their associated profiles (Figure 2).

This extension can be further used to handle any other type of trees associated to numerical matrices, such as phylogenetic profiles or protein classification results.

## Discussion

Most current tools for tree analysis are standalone applications focused on the visual inspection of trees. However, genomic studies often require the design of specific tests, or to program custom tasks over large collections of data. The Environment for Tree Exploration provides a programming framework to help in the implementation of specific analyses or visualization tasks. ETE's libraries can be imported together with other python toolkits in order to extend the functionality of tree handling operations. Furthermore, using the appropriate python modules, ETE can be connected to other environments such as R (using Rpy [25]), or BioPerl (using PyPerl [26]).

To date, ETE has been used in the automated phylogenomic pipelines employed to reconstruct and analyze complete phylomes [15,16,27]. Particularly, the use of ETE has enabled the application of phylogeny-based methods to predict orthology relationships on a genome wide scale [15]. Such approach is closer to the original definition of orthology and it is considered more accurate than classical, pair-wise based orthology prediction methods [28]. In addition, ETE has been used to implement a functional inference pipeline that enabled annotating more than 4,000 genes of the newly sequenced pea aphid genome [29]. This is the first case that such a reliable functional annotation pipeline has been applied to a newly sequenced genome. In addition, as implemented in the phylogenetic extension, ETE allows the use of phylogenetic trees to detect and date gene duplication events, a method that overcomes many of the known drawbacks of methods that are only based on sequence comparisons. Furthermore, ETE is used to provide interactive images of phylogenetic trees in projects such as PhylomeDB [16] and Phylemon [18], and to help in the interpretation of microarray clustering results as implemented in the Gene Expression Pattern Analyses Suite (GEPAS) [17]. Thus, ETE has already proved to be a useful resource in many different applications. By making this tool publicly available we expect that other projects can benefit from it and that ETE continues its expansion through the implementation of additional methods and features. ETE is under continuous development, with new modules and functionalities planned for future releases. We are also open to the possibility of including extensions from the open source community.

## Conclusions

ETE meets the needs of large-scale analyses of hierarchical tree data structures. It has been devised as a highly extensible and programmable toolkit, which has already been used in many different genomic and post-genomic projects. Future releases will incorporate novel analytical methods and extensions, some of which may come from the open source community.

A comprehensive tutorial, a programming guide including the main ETE package structure, and many basic examples are available from the 'Documentation' section at http://ete.cgenomics.org. Some practical examples are also available from the 'Examples' section at the same web site.

## Availability and requirements

Project name: ETE

Project home page: http://ete.cgenomics.org

Operating system(s): GNU/Linux

Programming language: Python

Other requirements: python-numpy, python-scipy, python-mysqldb, python-qt4

License: GNU GPL 3

## Authors' contributions

JHC implemented the toolkit and participated in designing the methods and in writing the manuscript. JD participated in designing clustering and visualization modules, and in writing the manuscript. TG participated in designing all phylogenetic methods and the phylomeDB API module, and in writing the manuscript.
